# The *Drosophila melanogaster Muc68E* Mucin Gene Influences Adult Size, Starvation Tolerance, and Cold Recovery

**DOI:** 10.1534/g3.116.029934

**Published:** 2016-04-25

**Authors:** Micael Reis, Ana C. Silva, Cristina P. Vieira, Jorge Vieira

**Affiliations:** * Instituto de Investigação e Inovação em Saúde, Universidade do Porto, 4200-135, Portugal; † Instituto de Biologia Molecular e Celular, Universidade do Porto, 4200-135, Portugal; ‡ Centre for Biomolecular Sciences, School of Life Sciences, University of Nottingham, NG7 2RD United Kingdom

**Keywords:** *Drosophila melanogaster*, starvation tolerance, cold resistance, mucin, *Muc68E*

## Abstract

Mucins have been implicated in many different biological processes, such as protection from mechanical damage, microorganisms, and toxic molecules, as well as providing a luminal scaffold during development. Nevertheless, it is conceivable that mucins have the potential to modulate food absorption as well, and thus contribute to the definition of several important phenotypic traits. Here we show that the *Drosophila melanogaster Muc68E* gene is 40- to 60-million-yr old, and is present in *Drosophila* species of the subgenus *Sophophora* only. The central repeat region of this gene is fast evolving, and shows evidence for repeated expansions/contractions. This and/or frequent gene conversion events lead to the homogenization of its repeats. The amino acid pattern P[ED][ED][ST][ST][ST] is found in the repeat region of Muc68E proteins from all *Drosophila* species studied, and can occur multiple times within a single conserved repeat block, and thus may have functional significance. *Muc68E* is a nonessential gene under laboratory conditions, but *Muc68E* mutant flies are smaller and lighter than controls at birth. However, at 4 d of age, *Muc68E* mutants are heavier, recover faster from chill-coma, and are more resistant to starvation than control flies, although they have the same percentage of lipids as controls. Mutant flies have enlarged abdominal size 1 d after chill-coma recovery, which is associated with higher lipid content. These results suggest that *Muc68E* has a role in metabolism modulation, food absorption, and/or feeding patterns in larvae and adults, and under normal and stress conditions. Such biological function is novel for mucin genes.

In *Drosophila* and many other insects, with the exception of Hemiptera and Thysanoptera, after feeding, the food bolus is surrounded by a structure called peritrophic matrix (PM), which is secreted by the cardia (a valve-like organ, at the junction between the foregut and the midgut) (Bolognesi *et al.* 2008). This matrix consists of a scaffold of chitin fibers embedded with glycosylated and, most often, chitin-binding proteins, named peritrophins (Lehane 1997). The PM is a physical barrier, lubricating the passage of food through the midgut and protecting its epithelium from pathogen invasion (Godl *et al.* 2002; Zhang *et al.* 2008; Hegedus *et al.* 2009). It also regulates nutrient uptake through the compartmentalization of digestive processes and prevents excretion of digestive enzymes by providing a means for enzyme recycling (Bolognesi *et al.* 2008). Furthermore, it serves as a biochemical barrier, sequestering, and, in some cases, inactivating, ingested toxins (Bolognesi *et al.* 2008). In addition to the PM, there is a mucous layer that lies between this matrix and the absorptive enterocytes, which also protects the midgut from mechanical damage, microorganisms, and toxic molecules, and must be kept hydrated and lubricated (Syed *et al.* 2008).

Our knowledge on the nature of the PM and the mucous layer is limited, but mucins have been found in their composition (Wang and Granados 1997; Syed *et al.* 2008). The main characteristic of mucin proteins is their extended regions of tandemly repeated sequences (PTS repeats), which contain prolines (P) together with serines (S), and/or threonines (T), which generally occupy between 30% and 90% of the protein length. Mucins also show signal peptides and it is well established that they are secreted into the lumen of the gut and Malpighian tubules, where they form enormous networks to which the glycosylated PTS repeats confer high water-binding capacity, a selective barrier function, and the ability to trap microorganisms (Hollingsworth and Swanson 2004; Hegedus *et al.* 2009). Besides this well-established role in protection against damage and microorganisms, mucins may also play a role during the organ morphogenesis of non chitin-producing organs by providing a luminal scaffold during their development (Syed *et al.* 2008).

In *D. melanogaster*, 15 *mucins* and eight *mucin*-like genes have been recognized (Syed *et al.* 2008), and six out of these 15 mucin proteins (Muc68E, Muc68D, Muc11A, Muc96D, Muc26B and Muc18B) show peritrophin A (PerA) chitin-binding domains. According to Flybase (http://flybase.org), *Muc96D*, *Muc26B*, and *Muc18B* are expressed almost exclusively in the larval midgut, *Muc68D* is expressed almost exclusively in the adult midgut, *Muc68E* is expressed in the adult and larval midgut, and *Muc11A* is expressed in the Malpighian tubules (both in adults and larvae) and the larval hindgut. Three of them (*Muc68D*, *Muc68E*, and *Muc18B*) show the highest expression in the Cardia/R1 region (Flygut database; http://flygut.epfl.ch/). The expression pattern of these three genes suggests that they may be peritrophins, and, thus, that they may influence, among other things, digestive efficiency.

It is possible that not all *D. melanogaster* mucins have been identified by Syed *et al.* (2008). For instance, the protein encoded by the *Frost* (*Fst*) gene shows a signal peptide, and 24.1% of the protein is composed by serines and threonines resembling a mucin (Colinet *et al.* 2010). Nevertheless, this protein was not identified as a mucin by Syed *et al.* (2008), because these authors used a 25% cut-off for the percentage of serines and threonines. Fst does not have a PerA chitin-binding domain, but only 26% of the proteins encoded by mucins and mucin-related genes show one (see above). This gene is expressed in mainly Malpighian tubules, although it is also expressed in the gut (Flybase.org; Chintapalli *et al.* 2007).

*Frost* has been implicated in the cold (Goto 2001; Sinclair *et al.* 2007; Colinet *et al.* 2010), desiccation (Sinclair *et al.* 2007), and immune response against virus, bacteria, and fungi (De Gregorio *et al.* 2001; Apidianakis *et al.* 2005; Chamilos *et al.* 2008; Buchon *et al.* 2009). Moreover, a strong negative correlation has been observed in *D. americana* between chill-coma recovery time (CCRT) and abdominal size (AS) (Reis *et al.* 2011). This is expected since, in *Drosophila*, temperature resistance is known to be dependent on body size (see, fon instance, van Heerwaarden and Sgro 2011). Therefore, proteins that may affect food absorption efficiency, such as mucins that are expressed in the midgut, may have an effect on adult size, and tolerance to starvation and cold. This would be a novel functional role for mucins. Moreover, Fst shows multiple evolutionary conserved “PEEST” motives (Goto 2001) (http://Flybase.org), and, together with Muc68E, are the only proteins in the *D. melanogaster* proteome with more than two “PEEST” motives (http://Flybase.org). Therefore, in this work, we address the possible role of Muc68E on adult size determination, starvation tolerance, and cold recovery.

The evolutionary analysis performed here for *Muc68E* shows that this gene is present only in the subgenus *Sophophora*. We also show that the middle repeat region of the protein is fast evolving, and that the amino acid pattern P[ED][ED][ST][ST][ST] (a generalization of the PEEST motif) is evolutionarily conserved, indicating that this sequence may have functional significance besides providing a sequence rich in prolines, serines, and threonines, typical of mucins. Furthermore, we show that the average lifespan (LS) of *D. melanogaster Muc68E* mutant female flies and their *w*^1118^ controls is not significantly different, indicating that this is a nonessential gene under laboratory conditions. This observation agrees with the lack of a *Muc68E* gene in the subgenus *Drosophila*. Nevertheless, at birth, *Muc68E* mutant female flies have smaller legs and wings, and are lighter than their *w*^1118^ controls, and, thus, the absence of a functional Muc68E protein has an impact on traits that are determined during the larval life phase. However, at 4 d of age, mutant flies recover faster from chill-coma (CC), and show increased tolerance to starvation (St). At 1 d after chill-coma recovery (CCR), mutant flies show bigger abdominal size (AS), which is associated with higher lipid content when compared to controls. In conclusion, *Muc68E* plays a role in metabolism modulation, food absorption, and/or feeding patterns, and thus contributes to adult size and weight, as well as to starvation and cold resistance.

## Materials and Methods

### Structural and evolutionary analyses

In order to study the evolution of the *Muc68E* gene, the relationships of the *Drosophila* species considered here (24 *Drosophila* species for which full genome data are available, namely *D. simulans*, *D. sechellia*, *D. melanogaster*, *D. erecta*, *D. yakuba*, *D. suzukii*, *D. biarmipes*, *D. takahashii*, *D. eugracilis*, *D. elegans*, *D. rhopaloa*, *D. ficusphila*, *D. kikkawai*, *D. bipectinata*, *D. ananassae*, *D. persimilis*, *D. pseudoobscura*, *D. miranda*, *D. willistoni*, *D. virilis*, *D. americana*, *D. mojavensis*, *D. grimshawi*, and *D. albomicans*) must be determined. *Muc68E* sequences may not be appropriate for this purpose because a large fraction of the protein (82% in *D. melanogaster*, for instance) is made of repeat sequence. The number of such repeats varies between species, and there is likely gene conversion between repeats (see *Results*), making any alignment of this region ambiguous. Therefore, besides showing a phylogeny for the *Muc68E* gene, we also determined the relationship of the *Drosophila* species here used based on the concatenation of a set of 16 highly conserved single copy genes that show no introns in *D. melanogaster* (*Ppox*, *CG32281*, *CG33230*, *CG12170*, *Rpn7*, *CG3570*, *eIF6*, *Prpk*, *CG14270*, *CG14512*, *Arpc4*, *Rpp20*, *CG33932*, *mRpL42*, *Bet1*, and *CG34117*). *D. melanogaster* gene sequences were obtained from Flybase (http://flybase.org). Genome sequences were downloaded from Flybase (http://flybase.org), or from NCBI (http://www.ncbi.nlm.nih.gov/), and manually annotated regarding the genes of interest, since many of the species here considered do not have an available genome annotation, or there is evidence that the provided annotation is wrong. Sequences from the 16 highly conserved genes were concatenated (in *D. melanogaster*, they represent 11,988 bp of sequence).

It should be noted that the *Muc68E* gene is intronless in *D*. *melanogaster*, and, thus, in principle, easy to annotate in the other species. Nevertheless, the repetitive nature of the central region of the gene means that it is prone to sequencing errors (especially insertion and deletions that cause out-of-frame changes) that lead to an incorrect annotation of the gene when automated gene annotation software is used. A tblastn approach was used to annotate this gene in all non-melanogaster species, using the Muc68E amino acid sequence as the query. In the case of *D. miranda*, *D. sechellia*, *D. elegans*, and *D willistoni*, one or more undetermined nucleotides were inserted in the sequence to put it back to the right frame. It should be noted that, in the case of *D. yakuba*, *D. miranda*, *D. persimilis*, *D. sechellia*, and *D. willistoni*, the region of the *Muc68E* coding sequence already contains one or more regions of undetermined sequence (Ns) that can be quite large, and in some cases put the *Muc68E* open reading frame out-of-frame. For *D. takahashii*, *D. suzukii*, *D. rhopaloa*, and *D. pseudoobscura*, no complete annotation of the *Muc68E* gene could be obtained, due to complex sequencing mistakes and/or missing sequence, although the tblastn search revealed an ortholog in these genomes (see *Results*). In Flybase (http://flybase.org), there are annotations available for the *Muc68E* ortholog in *D. simulans*, *D. sechellia*, *D. erecta*, *D. yakuba*, *D. ananassae*, and *D. willistoni*, Nevertheless, in every case there are reasons to believe that the provided annotation is likely wrong. Briefly, in *D. simulans*, the *GD12757* gene is given as the ortholog of *Muc68E*, but a blastn search of all annotated *D. simulans* genes, using the *D. melanogaster Muc68E* as query, reveals a single gene hit, namely *GD28325*. Moreover, a blastn search of all annotated *D. melanogaster* genes using the *D. simulans GD28325* gene as query reveals a single gene hit, namely *Muc68E*. The *GD28325* gene is annotated as having one intron in the coding region. This 443-bp intron shows 87% identity with the *D. melanogaster Muc68E* coding sequence over 99% of its length. In *D. sechellia*, the gene that is given as ortholog of *Muc68E* is the *GM24687* gene. The Flybase annotation includes a 528-bp DNA fragment that is not included in our annotation due to the presence of two putative introns in the former. When this 528 bp DNA sequence is blasted against all available *D. melanogaster* gene annotations, significant similarity is observed with the *CG42397* gene only. On the other hand, in our annotation, there is a 1812 bp fragment that is not included in the Flybase *GM24687* gene annotation. There is, however, one *D. sechellia* cDNA sequence (DK310984) that supports the coding potential of this 1812 bp fragment. When the cDNA DK310984 sequence is blasted (blastn) against all *D. sechellia* available gene annotations, no significant hits are obtained, showing that it is not included in any gene annotation. In *D. erecta*, the gene that is given as the *Muc68E* ortholog is *GG13865*. This gene is annotated as having one intron that is 474 bp long. This intron shows 89% identity over 88% of its size with the coding sequence of the gene, and thus is likely coding. In *D. yakuba*, the gene *GE20156* is given as the *Muc68E* ortholog. Our annotation and the Flybase annotation differ only by the presence of two putative short introns in the latter. One of the putative introns is almost identical to the coding region of gene *GE20156*, which is expected if the intron is wrongly annotated, since it is located in the middle of the highly repetitive region of the gene. In *D. ananassae*, the gene that is given as the ortholog of *Muc68E* is *GF24494*. The presence of two putative introns at the beginning of the *GF24494* gene implies the addition of a 645 bp fragment before the start of our annotation. When this 645 bp fragment is blasted (tblastx) against all *D. melanogaster* gene annotations, the first hit is with gene *CG42397* and not with *Muc68E*. There is a third intron in the *GF24494* gene annotation that is almost identical to the predicted coding sequence of the *GF24494* gene. As mentioned above, this is expected if the region is coding, since the putative intron is located in the middle of the highly repetitive region of the gene. In *D. willistoni*, the ortholog of *Muc68E* is *GK17254*, with three predicted introns. The first intron is 340 bp long, of which 335 bp are unknown sequence (Ns), and thus it is impossible to verify whether this putative intron shows protein coding features or not. The second and third introns (567 and 81 bp long respectively) show 96% identity with predicted coding regions for the gene *GK17254*, as expected if they are not intron sequence, since they are located in the middle of the repetitive region of the gene. For species of the *Drosophila* subgenus, no *Muc68E* orthologs could be found (see *Results*). In order to show that the nonidentification of *Muc68E* gene sequences in species of the *Drosophila* subgenus is not due to a high rate of divergence of this gene, we also performed a blastx search, using Muc68E as the query and, as database, the predicted coding sequences for *Drosophila* species for which there is an annotation, and retrieved all gene sequences showing a hit.

For all datasets, sequences were aligned using the ClustalW2 alignment algorithm as implemented in ADOPS (Reboiro-Jato *et al.* 2012). When this software is used, nucleotide sequences are first translated and then aligned using the amino acid alignment as a guide. Only codons with a support value above 2 are then used for phylogenetic reconstruction. Phylogenetic trees are obtained using MrBayes 3.1.2 (Ronquist *et al.* 2012), using the generalized time-reversible (GTR) model of sequence evolution, allowing for among-site rate variation and a proportion of invariable sites. Third codon positions are allowed to have a gamma distribution shape parameter different from that of first and second codon positions. Two independent runs of 2,000,000 generations with four chains each (one cold and three heated) are carried out. Convergence is assessed by looking at the average SD of split frequencies (that is ∼0.001), and at the potential scale reduction factor for every parameter (that was ∼1.00). Trees were sampled every 100th generation, and the first 5000 samples were discarded (burn-in). The remaining trees were used to compute the Bayesian posterior probabilities for each clade of the consensus tree. Synteny data were obtained using both the data available at Flybase (http://flybase.org), and by performing tblastn analyses of the region of interest against the *D. melanogaster* proteome. Amino acid logos were obtained using WebLogo (http://weblogo.berkeley.edu/logo.cgi).

The concatenated sequences of the 16 highly conserved genes used for the phylogenetic reconstruction analyses of the 24 *Drosophila* species for which there is an available genome, is provided in Supplemental Material, File S1 (FASTA format). The manual annotation of the *Muc68E* gene in the species of the *Sophophora* subgenus, based on the protein sequence of *D. melanogaster*, is provided in File S2 (FASTA format), the *Muc68E* coding sequence alignment used in the phylogenetic analyses is provided in File S3 (FASTA format), and the coding sequence alignment used in the mucin phylogenetic analyses is provided in File S4 (FASTA format).

### Gene expression

In order to determine the effect of the *Muc68E P*-element insertion on the *Muc68E* gene expression, two primer pairs were designed for both the region upstream (forward primer: GCACAAAGCACAGGTATCAT, and reverse primer: ACTACTGTCACCGCAAC) and downstream (forward primer: TCCCCTGAAACCACAACTT, and reverse primer: AAACACCTGAATCTCCACTGC) of the transposable element insertion. The gene *EF1a-48D* (forward primer: AAGACCACCGAGGAGAACCC, and reverse primer: CAGCGAAGCGACCCAGAG) was used as a positive control. We confirmed that these primers have a unique pairing sequence using BLAST search. Flies from strains 5905 (*w*^1118^) and 27851 (*w*^1118^*Muc68E*) were snap-frozen in liquid nitrogen and stored at –80° until mRNA extraction. Total RNA was isolated from whole bodies using TRIzol Reagent (Invitrogen, Spain) according to the manufacturer’s instructions, and treated with Turbo DNA-free kit (Life technologies, Carlsbad, CA). The purity and concentration of the extracted samples was measured with NanoDrop ND-1000 spectrophotometer (NanoDrop, Thermo Scientific, Portugal), and RNA integrity was checked using Experion platform (Bio-Rad, Portugal; all the samples had high RQI values). cDNA was synthesized by reverse transcription of 1.0 μg of RNA of each sample with SuperScript III First-Strand Synthesis SuperMix for qRT-PCR (Invitrogen, Spain) using random primers. Reactions where template was not added, and reactions with RNA that was not reverse transcribed, were performed to confirm the absence of genomic DNA contamination.

### Phenotypic characterization

Female flies of *D. melanogaster* strains 5905 (*w*^1118^) and 27851 (*w*^1118^*Muc68E*) were phenotyped for lifespan (LS), starvation (St), chill-coma recovery time (CCRT), and body size. Only females were used to minimize the possible effect of sex on the results. Fly stocks were maintained in vials containing standard food under low density conditions at 25°, and under 12 h of light and 12 h of dark (12L:12D) cycles. At least 100 female flies from each strain were collected within 8 hr after eclosion in order to guarantee that they were virgins. The phenotypes were addressed in 4-d-old adult flies to reduce the influence of age on the results.

In order to measure LS, flies were kept in individual vials at 25° under 12L:12D cycles. Their condition was checked every other day, and vials were changed every week until they died. To address tolerance to St, mortality curves were determined for flies kept in vials containing cotton soaked with water. These experiments were conducted with sets of five female flies in each vial at 25° under 12L:12D cycles, and their status was checked every 12 hr until they all died. For cold shock, individual flies were transferred to empty vials, which were then sealed with Parafilm, and buried in ice. After 4 hr of cold exposure at 0°, we measured individual CCRT at 25°. Flies were considered to be recovered from chill-coma when they were able to stand up on all their legs [for more details on the procedure used see Reis *et al.* (2011)]. At 1 d after recording CCRT, each fly was individually photographed using a stereomicroscope Nikon ZMS 1500 H with a magnification of 20 ×. Then, one leg of each of the three pairs, and one wing of each fly was dissected and mounted on a slide with Hoyer’s medium. Individual pictures of each structure were captured using the stereomicroscope with magnifications of 50 × and 40 × for legs and wings, respectively. The resulting JPG files were saved with a resolution of 1600 × 1200 pixels and the pictures were treated using ImageJ (http://imagej.nih.gov/ij/; Schneider *et al.* 2012) in order to enhance the contrast by increasing pixel saturation in 0.4 before measuring tibia lengths, as well as abdominal and wings areas. A ruler was also photographed, under the same conditions, to allow for the conversion between number of pixels and mm or mm^2^.

### Lipid content

In order to determine the impact of the absence of Muc68E protein on lipid content, we determined the lipid weight of female flies at different ages (0 d; 4 d; 5 d). The protocol used was adapted from Parkash *et al.* (2012), and at least nine sets of 10 female flies were used for each age. The samples were dehydrated at 65° for at least 24 hr and their dry weight (DW) was determined using a microscale Sartorius (precision of 0.01 mg). Then, the samples were transferred into 15-mL tubes and 5 mL of diethyl ether was added in order to dissolve the lipid content during 72 hr at room temperature with constant agitation. The samples were subsequently dried at 65° for at least 24 hr, and their weight was again recorded [lipid-free dry weight (L-free DW)]. Total lipid content (LW) was determined by subtracting the L-free DW from the DW.

### Data availability

The authors state that all data necessary for confirming the conclusions presented in the article are represented fully within the article.

## Results

### Muc68E structural features and evolution

**Figure 1:**
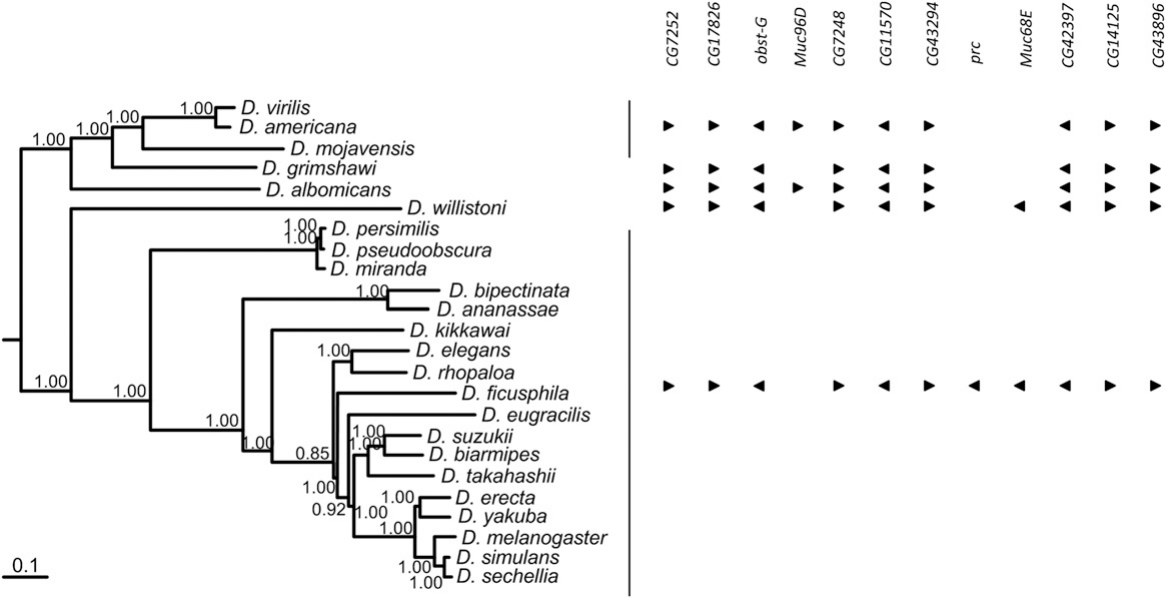
Phylogenetic relationships of 24 *Drosophila* species with available genomes based on the sequences of 16 genes (numbers near the nodes are posterior credibility values), and analysis of *Muc68E* distribution across the *Drosophila* genus, as well as the synteny map of the region surrounding this gene, not drawn to scale. In *D. melanogaster*, it corresponds to region 3L:11947675..11983764 (http://flybase.org).

When 24 Drosophila species for which a genome sequence is available (*D. simulans*, *D. sechellia*, *D. melanogaster*, *D. erecta*, *D. yakuba*, *D. suzukii*, *D. biarmipes*, *D. takahashii*, *D. eugracilis*, *D. elegans*, *D. rhopaloa*, *D. ficusphila*, *D. kikkawai*, *D. bipectinata*, *D. ananassae*, *D. persimilis*, *D. pseudoobscura*, *D. miranda*, *D. willistoni*, *D. virilis*, *D. americana*, *D. mojavensis*, *D. grimshawi*, and *D. albomicans*) are considered, an ortholog of *Muc68E* gene can be found in species of the subgenus *Sophophora*. Figure S1 and Figure S2 show that the *Muc68E* gene was correctly identified. Indeed, phylogenetic analysis of the *Muc68E* coding sequences not showing ambiguous positions (Figure S1) recapitulates the species relationships obtained using a dataset of 16 highly conserved single copy genes, that in *D. melanogaster* show no introns and thus are easily annotated in any genome (see Figure 1). Moreover, when all genes that show a hit in a blastx search, using Muc68E as the query and the predicted coding sequences as database, the resulting phylogenetic tree shows that all *Muc68E* sequences (only those without unambiguous positions were used) cluster together, and that two paralogous genes (*CG42397* and *CG17826*; Figure S2) are identified. Therefore, the failure to identify *Muc68E* gene in species from the *Drosophila* subgenus is not an artifact of the BLAST methodology used, since paralogous *mucin* genes are identified. It should be noted that there are several structural features that are observed only in Muc68E proteins and that are not found in the predicted protein set for species of the *Drosophila* subgenus (see below). Although the *Drosophila* subgenus is represented by five species only (*D. virilis*, *D. americana*, *D. mojavensis*, *D. grimshawi*, and *D. albomicans*), these species are highly divergent (Morales-Hojas and Vieira 2012; Russo *et al.* 2013), and, thus, it is very likely that *Muc68E* is missing in all species of this subgenus. It should be noted that, although *Muc68E* is missing in the species of the *Drosophila* subgenus, the microsynteny of the *D. melanogaster* 3L:11947675…11983764 (http://flybase.org) region is largely conserved (Figure 1). The *Muc96D* gene is, however, present in the region in between genes *CG7252* and *CG43896*, in *D. americana*, *D. virilis*, *D. mojavensis*, and *D. albomicans* (species from the *Drosophila* subgenus), but is elsewhere in the genomes of the *Sophophora* subgenus species. For instance, in *D. melanogaster*, *Muc96D* is located on another Muller’s element within an intron of the *Fur1* gene (http://flybase.org). Also, in *D. grimshawii*, this gene was apparently lost. Moreover, in the region in between genes *CG7252* and *CG43896*, the *pericardin* (*prc*) gene was inserted after the separation of *D. willistoni* lineage (a *prc* gene can be found in all species in a different region of the genome). This evolutionary event is not, however, correlated with the appearance of the *Muc68E* gene, since in *D. willistoni*, a *Muc68E* gene is detected in this region, but the *prc* gene is located elsewhere in the same chromosome arm (http://flybase.org). The lack of a *Muc68E* gene in species of the *Drosophila* subgenus suggests that *Muc68E* is not an essential gene. Using the same methodology, no *Muc68E* ortholog could be found in non-*Drosophila* insects, such as *Culex quinquefasciatus* and *Anopheles gambiae* (data not shown).

For the remaining analyses we used only the species for which a complete annotation of the *Muc68E* gene could be obtained (see *Materials and Methods*). Muc68E is a mucin (Syed *et al.* 2008), and, thus, it should harbor a clear signal peptide in order to be exported to the extracellular space. Nevertheless, no signal peptide is predicted when the *D. melanogaster* Muc68E protein and the Signal IP server (http://www.cbs.dtu.dk/services/SignalP/) is used. However, at amino acid position 24, there is another methionine, which when assumed to be the translation start—a peptide signal is predicted in every *Drosophila* species analyzed. The *D. melanogaster* methionine 24 is conserved in all *Drosophila* species analyzed, and in some species, particularly *D. elegans*, *D. ananassae*, *D. bipectinata*, *D. biarmipes*, *D. miranda*, *D. kikkawai*, and *D. willistoni*, there is no other methionine that could be used as the translation start. Therefore, it is likely that this methionine is the true start site of this protein in every *Drosophila* species.

The *D. melanogaster* Muc68E protein shows a large number of “PEEST” motives that could have functional importance. It should be noted that this is a rare motif in the *D. melanogaster* proteome, and that only this protein and Frost (Fst) show more than two “PEEST” motives. Frost shows a signal peptide, and 24.1% of this protein is composed by serines and threonines, and, thus, resembles a mucin (Colinet *et al.* 2010).

**Figure 2:**
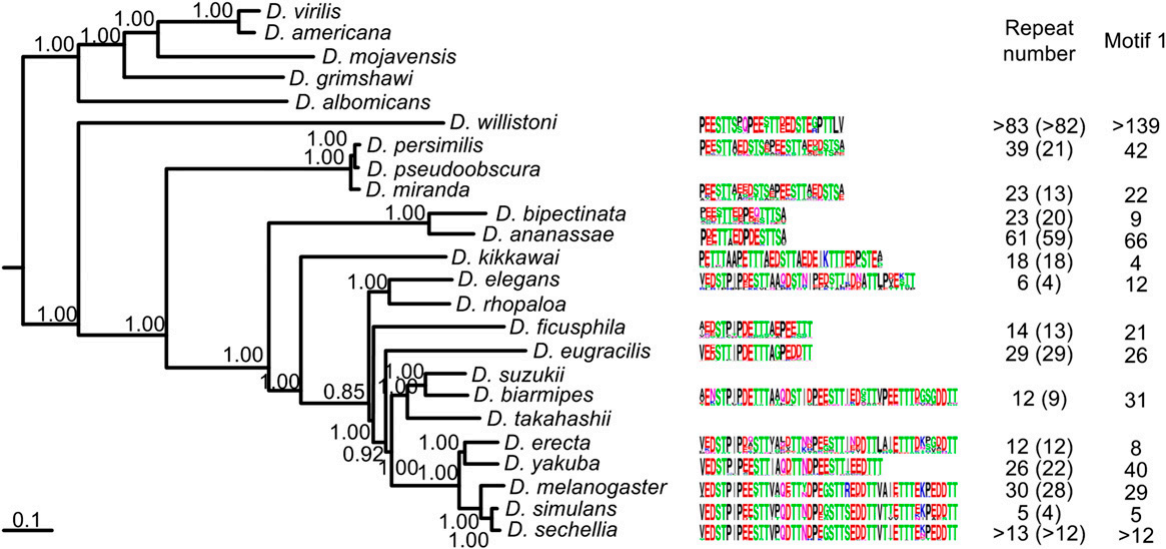
Phylogenetic relationships of 24 *Drosophila* species with available genomes based on the sequences of 16 genes (numbers near the nodes are posterior credibility values), and amino acid Web logos analysis of Muc68E highly repetitive region across *Drosophila* genus. Muc68E is present only in species of the *Sophophora* subgenus. However, due to complex sequencing mistakes, and/or missing sequence, a confident complete annotation of *Muc68E* was not possible for *D. takahashii*, *D. suzukii*, *D. rhopaloa*, and *D. pseudoobscura*.

Not all species that could be analyzed here have “PEEST” motives (Figure 2), and in all of them, the size of conserved blocks is much larger than the “PEEST” motif. Conserved repeat blocks are easy to recognize because, for most species, there is little variation between repeated blocks. The size of the conserved repeat block varies greatly (from 16 to 48 amino acids). This is different from what happens with Frost, where “PEEST” motifs are not included in larger conserved blocks.

The number of conserved repeat blocks varies greatly as well (from five up to more than 82 repeats; Figure 2). It should be noted that the number of repeats may be underestimated because highly degenerated repeats are not easy to recognize. Although the amino acid logos are, for most species, very homogeneous, there are fixed differences between species, even between closely related species, such as *D. melanogaster* and *D. simulans* (see, for instance, the fixed difference at repeat positions 28 and 35 in Figure 2). This implies either a significant amount of within-gene conversion within the central region of the gene, and/or frequent contractions and expansions of the repeats. The amino acid pattern P[ED][ED][ST][ST][ST] is common to all species, and can occur multiple times within a single conserved repeat block (pattern 1 in Figure 2). The conservation of pattern 1 in the proteins encoded by *Muc68E* along the *Drosophila* phylogeny, despite the evidence for multiple contractions/expansions of the middle part of the gene and/or frequent gene conversion events within the gene, suggests that this sequence may have functional significance besides providing a sequence rich in prolines, serines, and threonines, typical of mucins.

In the *D. melanogaster* proteome, besides *Muc68E* and *Fst*, there are only two more genes (*Mur18B* and *CG6296*) encoding proteins where pattern 1 is present more than twice. *Mur18B* is a mucin-related gene that shows high levels of expression in the Malpighian tubules of larvae and adult flies, while *CG6296* is a lipase that is expressed exclusively in the midgut of larvae and adults (http://FlyBase.org). Therefore, *D. melanogaster* proteins with more than two pattern 1 motives are rare, and are observed in genes that are almost exclusively expressed in the gut (*Muc68E*, *CG6296*), Malpighian tubules (*Mur18B*), or both (*Fst*). Given these results, we can say that pattern 1 is rare within mucin and mucin-related proteins, since it is present in only two (*Muc68E* and *Mur18B*) out of the 23 genes identified by (Syed *et al.* 2008) as belonging to this class. It should be noted that Colinet *et al.* (2010), already detected some amino acid sequence similarity between Fst and the proteins encoded by two mucin and mucin-related genes, namely, *Muc11A* and *Mur18B*.

Muc68E shows three chitin-binding domains in the C-terminal region, with a typical CX_2_GX_9_CX_5_CX_9_CX_5_WX_6_CX_6_C motif that can be identified as a peritrophin-A (PerA) domain, which is more commonly called chitin-binding type-2 domain (CBT2 domain) (Gaines *et al.* 2003). It should be noted that the proteins encoded by all genes in the *CG7252* and *CG43896* region, except *prc*, have CBT2 domains similar to the ones in Muc68E, which follow the more general PerA motif (CX_13–20_CX_5–6_CX_9–19_CX_10–14_CX_4–14_C), but the CX_2_GX_9_CX_5_CX_9_CX_5_WX_6_CX_6_C motif is found only in the *Muc68E* protein. When pattern hit initiated blast (PHI blast) is performed using the *D. melanogaster* Muc68E protein as the query, against all sequences deposited at the NCBI protein database, significant hits are observed only with the Muc68E proteins from species of the subgenus Sophophora, and the mucin-2-like sequence from *Stomoxys calcitrans* (Muscidae). However, the latter putative protein does not have the repeats that are typical of Muc68E, and presents 11 CBT2 domains, rather than the three that are always observed in Muc68E. *Muc68E* may have arisen from the duplication and expansion of the repeat region of one neighboring gene, although the alternative hypothesis that it is an old gene that has been lost in the subgenus *Drosophila* cannot be ruled out, since the highly specific amino acid pattern CX_2_GX_9_CX_5_CX_9_CX_5_WX_6_CX_6_C has been found in Muscidae. Nevertheless, these genes are too divergent to confidently reconstruct its evolutionary history (data not shown).

### Muc68E is not an essential gene but affects adult weight at birth

**Figure 3:**
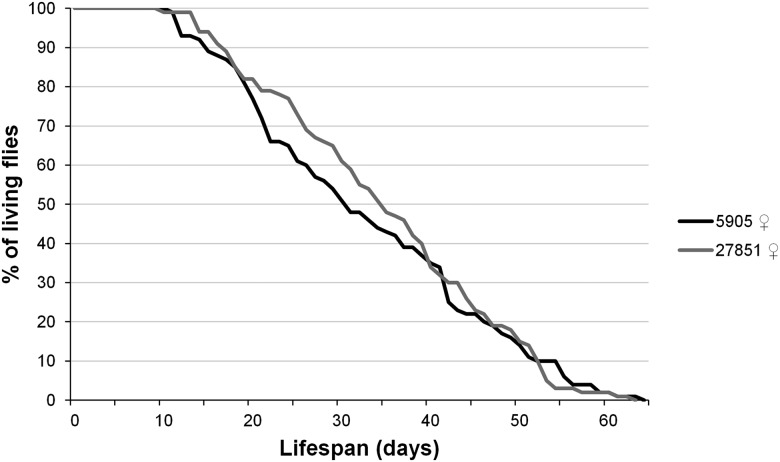
Survival curves over time. For *D. melanogaster* strains 5905 (*w*^1118^) and 27851 (*w*^1118^Muc68E), 100 females were reared at 25° under 12L:12D cycles in vials with standard food, and their condition was checked every other day, while the vials were changed every week until they died. The average lifespan for 5905 and 27851 is 33.9 ± 14.0 and 35.9 ± 13.0, respectively.

**Figure 4:**
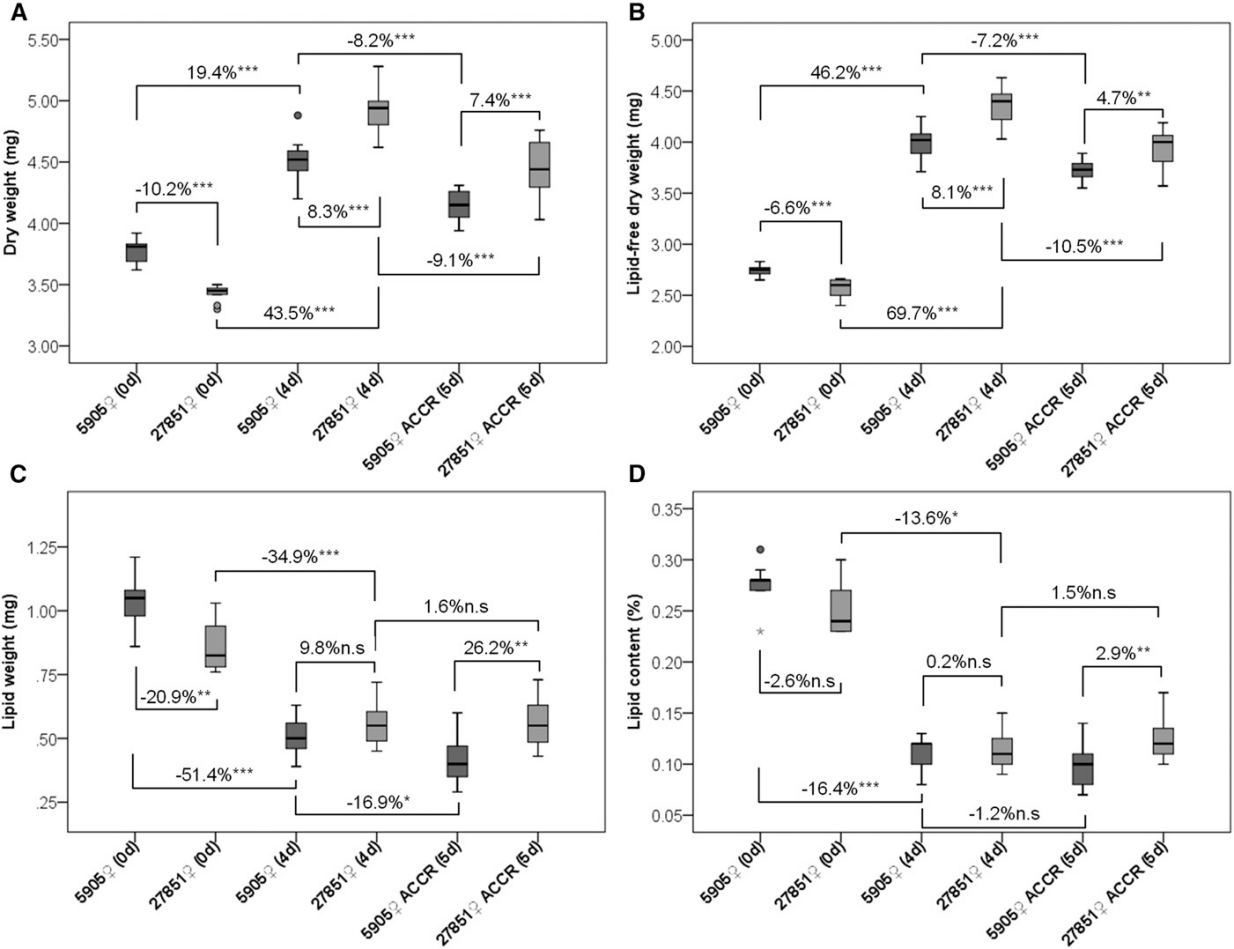
Changes in total body weight and lipid content over time between 27851 (*w*^1118^Muc68E) mutants and 5905 (*w*^1118^) controls. 0d, newborn flies; 4d, 4-d-old flies; 5d, 5-d-old flies; ACCR, after chill coma recovery. For each strain and age, at least nine sets of 10 females were dehydrated and their dry weight (DW) assessed (A). The lipid content was then dissolved with diethyl ether and their lipid-free dry weight (L-free DW) determined (B). Lipid weight (LW) was calculated by subtracting L-free DW from DW (C). Lipid content represents the percentage of DW composed by lipids (D). The differences in percentage for the averages of all weighing measurements between mutants and controls, and between different ages for each strain, are shown with their respective significance (n.s. *P* > 0.05; * *P* < 0.05; ** 0.01 > *P* > 0.001; *** *P* < 0.001).

Survival curves throughout time were determined for 5905 (*w*^1118^) and 27851 (*w*^1118^Muc68E) females (Figure 3). Female flies of strains 5905 and 27851 live, on average, 33.9 and 35.9 d, respectively, and this difference is not statistically significant (nonparametric Mann-Whitney test, *P* > 0.05; Figure 3). These strains are genetically identical with the exception of the 7.5 kb *P*-element insertion at the beginning (405 bp downstream of the first methionine according to the Flybase annotation) of the *Muc68E* coding region. Primers designed for the sequence that precedes the transposable element insertion support the amplification of a DNA fragment of the predicted size when using cDNA from strain 5905 and 27851, while primers designed for the sequence downstream of the transposable element insertion support the amplification of a DNA fragment of the predicted size when using cDNA from strain 5905 only. Therefore, the *P*-element insertion does not abolish the transcription of the *Muc68E* gene but leads, as expected, to the premature termination of the transcript. It is unclear whether a stable protein is produced, although it seems unlikely since the resulting protein would have about 7.5% of the normal size and would miss entirely the central repeat region and the chitin binding domains. Therefore, under laboratory conditions, the absence of a functional Muc68E protein does not seem to compromise any essential aspect of fly physiology. It could be argued that mucins are a large gene family, and that the function of *Muc68E* might be replaced by another member, but unfortunately there is no phenotypic information for any other *Drosophila* mucin. Nevertheless, a detailed analysis shows that there are differences between *Muc68E* mutants and controls, and, thus, if true, the *Muc68E* function is only partially replaced by other mucins. Indeed, mutant newborn flies are significantly lighter than controls, showing less 10.2% of DW (nonparametric Mann-Whitney test, *P* < 0.001; Figure 4A), implying that *Muc68E* has a role in larval metabolism, food absorption, and/or feeding pattern. In these flies both the L-free DW (–6.6%, nonparametric Mann-Whitney test, *P* < 0.001; Figure 4B), and the LW (–20.9%, nonparametric Mann-Whitney test, *P* < 0.001; Figure 4C) are also significantly lower in mutants, but the percentage of DW composed by lipids is not significantly different from controls (nonparametric Mann-Whitney test, *P* > 0.05; Figure 4D). Therefore, the reduction in weight in mutants does not imply a reduction in the percentage of lipid reserves.

### Four-d-old Muc68E mutant flies are heavier and recover faster from cold shock

**Figure 5:**
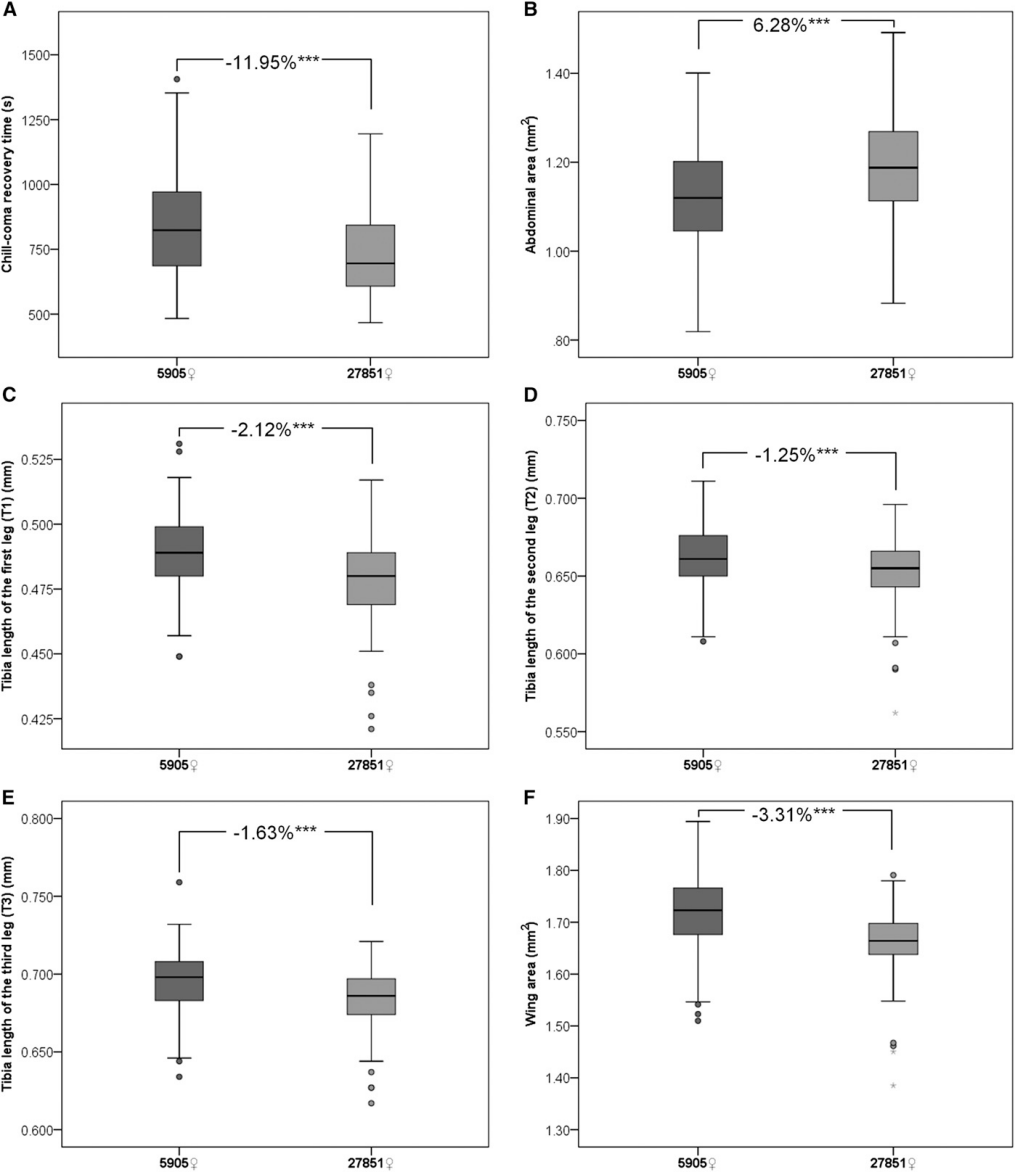
Boxplot showing the differences in cold tolerance and body size between female flies of the control strain 5905 (*w*^1118^) and the *Muc68E* mutant strain 27851 (*w*^1118^*Muc68E*). Outliers are shown as circles, and extreme outliers as stars. Individual chill-coma recovery times (CCRT) were determined for 122 mutant female flies and 162 controls. (A) Individual pictures of each female were taken and the abdominal area was determined using ImageJ (B). The same individuals were subsequently dissected, and the tibia lengths of one leg of each pair (C)–(E), as well as the area of one wing (F) were addressed using ImageJ. The differences in percentage for each phenotype measured between mutants and controls are shown, and all comparisons are highly significant (*** *P* < 0.001).

Muc68E is a mucin, and very likely a peritrophin that affects weight at birth (see above). Moreover, Muc68E shows the evolutionarily conserved amino acid pattern 1 that is also observed in the protein encoded by *Fst* (see above), a gene that has been implicated in cold tolerance (Goto 2001; Sinclair *et al.* 2007; Colinet *et al.* 2010; Reis *et al.* 2011). Given these observations, we hypothesized that Muc68E could influence the response and recovery from different stresses, including rapid exposure to cold. Therefore, we compared the CCRT obtained for 4-d-old control (5905) and mutant (27851) female flies. There is a highly significant statistical difference between strain 5905 and strain 27851 regarding CCRT (nonparametric Mann-Whitney test; *P* < 0.001). Flies without a functional Muc68E protein (27851) recover on average 12.0% faster than control flies (5905) (Figure 5A). It should be noted that, when compared with newborn flies, 4-d-old flies show a dramatic decrease in the amount of lipids, which goes along with an increase in DW and L-free DW (Figure 4, A–C). Surprisingly, 4-d-old mutant females are now significantly heavier than their controls (8.3%; Mann-Whitney test, *P* < 0.001; Figure 4A), despite the percentage of lipids not being significantly different between the two strains (nonparametric Mann-Whitney test, *P* > 0.05; Figure 4, C–D). Therefore, adult flies are consuming their lipid reserves to increase their weight, although food intake must also play an important role. These results suggest that the presence of a functional Muc68E protein has an opposite effect on weight determination during developmental and adult stages, and that *Muc68E* has a role in adult metabolism, food absorption, and/or feeding pattern, as well.

Given the negative correlation previously reported between AS and CCRT (Reis *et al.* 2011), and the observation that 4-d-old mutant females are heavier than their controls, we determined the AS of all individuals subjected to CC, 1 d after being exposed to cold stress. Female flies from the mutant strain 27851 show 6.3% bigger abdomens than control females from strain 5905 (Figure 5B), and this difference is statistically significant (nonparametric Mann-Whitney test; *P* < 0.001). As expected, the strain showing bigger AS recovers faster from CC. Nevertheless, the observed increase in cold tolerance cannot be explained by differences in the percentage of lipids in 4-d-old flies (Figure 4D).

A significant negative correlation between AS and CCRT was observed for 27851 strain (*N* = 122; Pearson’s ρ = –0.181, *P* = 0.046; Spearman’s ρ = –0.160, *P* = 0.079), but not for 5905 (*N* = 162, Pearson’s ρ = –0.074; *P* = 0.350; Spearman’s ρ = –0.048, *P* = 0.545). When all data are used, the negative correlation remains significant, and the amount of variation in CCRT explained by AS is similar to that explained by the data for 27851 alone (*N* = 284; Pearson’s ρ = –0.186, *P* = 0.002; Spearman’s ρ = –0.163, *P* = 0.006). This implies that the intrastrain variability in CCRT, is explained to a small extent by variation in AS.

Despite the larger AS observed for mutants when compared to controls 1 d after CC, mutant females show significantly smaller legs and wings than controls (Figure 5, C–F, Mann-Whitney test; *P* < 0.001). This result was expected, since mutant flies are lighter than controls at birth (Figure 4A), and legs and wings do not grow after adult eclosion (*e.g.*, Nijhout 2003; Edgar 2006). The differences in size between legs vary in between 1.3% and 2.1% (Figure 4, C–E) and the wings of 27851 females are 3.3% smaller than the wings of 5905 controls (Figure 4F).

Within strains, CCRT is significantly and negatively correlated with both the tibia length of the third leg (T3) (most posterior pair) (Pearson’s ρ = –0.235, *P* = 0.009; Spearman’s ρ = –0.223, *P* = 0.014 for 27851, and Pearson’s ρ = –0.232, *P* = 0.003; Spearman’s ρ = –0.207, *P* = 0.008 for 5905), and the area of the wing (Pearson’s ρ = –0.190, *P* = 0.037 Spearman’s ρ = –0.111, *P* = 0.222 for 27851, and Pearson’s ρ = –0.241, *P* = 0.002; Spearman’s ρ = –0.230, *P* = 0.003 for 5905). Size differences in these anatomical structures explain more variation in CCRT than AS within each strain. Nevertheless, when all data are analyzed, the correlation between wing size and CCRT becomes nonsignificant (Pearson’s ρ = –0.091, *P* = 0.127 Spearman’s ρ = –0.050, *P* = 0.402), and the amount of variation in CCRT explained by T3 is less than that explained by AS (Pearson’s ρ = –0.139, *P* = 0.019; Spearman’s ρ = –0.125, *P* = 0.035 and Pearson’s ρ = –0.186, *P* = 0.002; Spearman’s ρ = –0.163, *P* = 0.006 for T3 and AS, respectively). Thus, within the strains, a small fraction of the variation in CCRT may be explained by differences in size determined during development, while between strains it may be explained by differences in size determined during adulthood, before or after CCRT.

Regarding weight and lipid content, 1 d after CCRT there is a 16.9% significant reduction of LW in the controls (nonparametric Mann-Whitney test, *P* < 0.05, Figure 4C), but not in the mutants (nonparametric Mann-Whitney test, *P* > 0.05; Figure 4C). Nevertheless, there is a remarkable reduction in DW in both strains (–8.2% and –9.1% for controls and mutants, respectively; nonparametric Mann-Whitney test, *P* < 0.001; Figure 4A). Therefore, it is likely that other metabolic components have been consumed in response to chill coma in both strains and some lipids in controls only, or that food absorption or feeding patterns were different after the exposure to the stress in the two strains. In *Drosophila*, it has been reported that glucose and trehalose levels increase in response to cold exposure (Overgaard *et al.* 2007), and, therefore, these molecules are likely used first, and only then lipids may be used to restore these sugars (Arrese and Soulages 2010).

**Figure 6:**
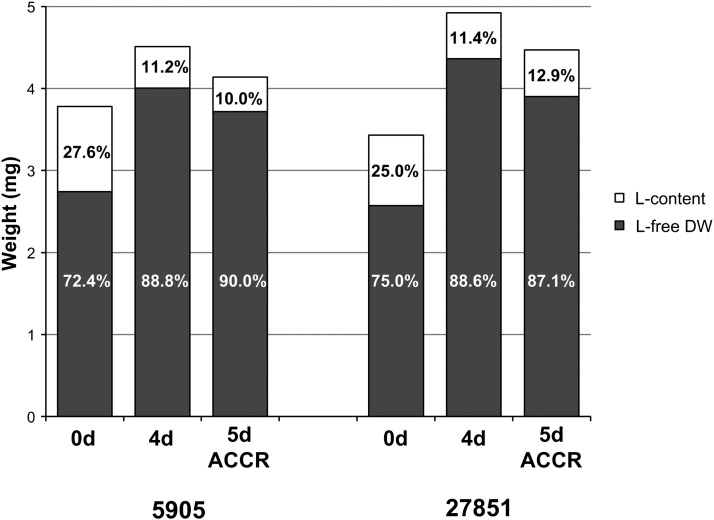
Summary of the variation in the mean values of total body weight and lipid content obtained for sets of 10 females throughout time between mutant (27851 *w*^1118^Muc68E) and control (5905 *w*^1118^) strains. 0d, newborn flies; 4d, 4-d-old flies; 5d, 5-d-old flies; ACCR, after chill coma recovery.

A summary of the changes in weight and lipid content over time in strains 5905 and 27851 is shown in Figure 6.

### Muc68E mutant female flies show increased survival under starvation-inducing conditions

The adult fat body is composed by adipose tissue filled with lipids, and it is located in the abdomen (http://Flybase.org). Therefore, the observation that 1 d after chill coma recovery, mutant flies have larger AS and show a higher percentage of lipids than controls, suggests that mutant flies use their lipid reserves differently than controls in response to, or to recover from, cold stress. Since it has been reported that increased fat content in *D. melanogaster* is positively associated with starvation (St) tolerance (Parkash *et al.* 2012), we decided to compare this phenotype in control and mutant flies.

**Figure 7:**
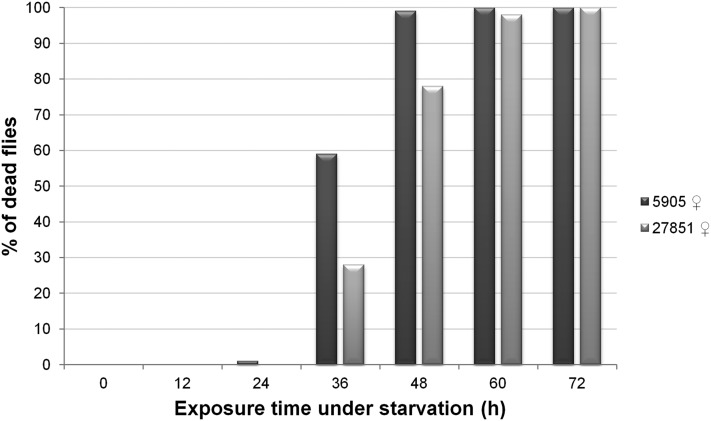
Mortality curves under starvation over time. For each *D. melanogaster* strains 5905 (*w*^1118^) and 27851 (*w*^1118^Muc68E), 100 females were reared at 25° under 12L:12D cycles in empty vials with cotton soaked in water, and their condition was checked every 12 hr until they died.

In order to determine how well control and mutant female flies are able to stand prolonged St, the percentage of living flies present in vials containing cotton soaked with water at 25° was recorded every 12 hr until all flies died. There is almost no mortality (less than 5%) in the first 14 hr, and no fly was able to stand the treatment for 72 hr (Figure 7). For both strains, the majority of flies die between 24 hr and 48 hr. Under starvation-inducing conditions, females of strain 5905 show a statistically significant increase of 31% in mortality after 36 hr (Fisher’s exact test; *P* = 1.6E-05), and 21% after 48 hr (Fisher’s exact test; *P* = 2.0E-06) when compared with mutant females. This result suggests that the lack of a functional Muc68E protein leads to increased resistance to this stress, probably due to a different use of the lipid reserves.

## Discussion

Mucins and peritrophins have been implicated in many different biological processes, such as protection against mechanical damage, microorganisms, and toxic molecules, as well as providing a luminal scaffold during development (Godl *et al.* 2002; Syed *et al.* 2008; Zhang *et al.* 2008; Hegedus *et al.* 2009). Nevertheless, the biological role of each mucin gene remains largely elusive.

*Muc68E* is present only in species of the *Sophophora* subgenus, and no ortholog has been found in non-*Drosophila* insects. Therefore, this gene is likely to be around 40–60 million yr old (Morales-Hojas and Vieira 2012; Russo *et al.* 2013). The protein encoded by *Muc68E* gene shows the two typical features of mucins, namely, the presence of a peptide signal (although this implies that the *D. melanogaster Muc68E* coding sequence is wrongly annotated in Flybase; see *Results*), and an extended region of tandemly repeated sequences that is enriched in prolines (P), serines (S), and threonines (T). This repeat region is fast evolving, and shows evidence for frequent contractions and expansions likely mediated by unequal crossing over events. These events, together with frequent within gene conversion lead to the homogenization of repeat blocks. Whether the observed repeat number and length variability is the result of selection remains to be elucidated. The amino acid pattern P[ED][ED][ST][ST][ST] is found in Muc68E proteins of all species studied, and can occur multiple times within a single conserved repeat block. Given the evolutionary conservation of this pattern despite the fast evolution of the gene structure, this sequence may have functional significance besides providing a sequence rich in prolines, serines, and threonines that is typical of mucins. The presence of three evolutionarily conserved PerA domains, and its expression pattern (mostly expressed in the Cardia/R1 region), suggests that this gene may also be a peritrophin.

*Muc68E* mutant and control flies have similar average lifespan, showing that, under laboratory conditions, this is a nonessential gene, as expected from the observation that there is no ortholog in the genome of species from the *Drosophila* subgenus. Nevertheless, because *Muc68E* is a mucin, the smaller size and weight observed for newborn mutant female flies when compared with controls likely implies that *Muc68E* has a role in larval metabolism, food absorption, and/or feeding pattern. Also, the reduction in weight does not imply a reduction in the percentage of lipid reserves.

In 4-d-old adults, however, the opposite effect is observed. Indeed, *Muc68E* mutants are heavier than controls, and this difference is not due to a differential accumulation of lipids. Therefore, *Muc68E* also has a role in adult metabolism, food absorption, and/or feeding pattern. Four-d-old *Muc68E* mutant flies are also more resistant to starvation, and recover faster from chill-coma than control flies. Moreover, when compared to controls, *Muc68E* mutant flies have enlarged abdominal size 1 d after CCR, which is associated with a higher lipid percentage. These results imply that mutant and control flies use their lipid reserves differently during, or after, these stresses.

Compatible with the view that *Muc68E* influences metabolism, food absorption, and/or the feeding pattern is the observation that *Drosophila* microbiota influences host energy homeostasis and carbohydrate allocation patterns in adults (Ridley *et al.* 2012), by promoting the expression of genes involved in host digestive functions and primary metabolism, but also of other “nonmetabolic genes” still related to metabolic activities, including the *Muc68E* gene (Combe *et al.* 2014).

In conclusion, *Muc68E* gene modulates metabolism, food absorption, and/or feeding responses both in larvae and adults, although differently. The large number of potential biological roles played by mucins means that this class of genes may often be the target of contradictory selective pressures, thus explaining the unexpected observation that *Muc68E* mutant flies perform better under a variety of stressful conditions than their controls.

## Supplementary Material

Supplemental Material
