# Supplementary material for: The Drosophila melanogaster Muc68E Mucin Gene Influences Adult Size, Starvation Tolerance, and Cold Recovery
Source: G3 (Bethesda). 2016 Apr 25;6(7):1841–51. doi: 10.1534/g3.116.029934 (PMC4938639; doi:10.1534/g3.116.029934)
Supplement: Supplemental Material [file supp_6_7_1841__index.html]

The Drosophila melanogaster Muc68E Mucin Gene Influences Adult Size, Starvation Tolerance, and Cold Recovery — Supplemental Material 

# The *Drosophila melanogaster Muc68E* Mucin Gene Influences Adult Size, Starvation Tolerance, and Cold Recovery

## Supplemental Material for Reis *et al.*, 2016

**Files in this Data Supplement:**

- Figure S1 - Phylogenetic relationships of the annotated *Muc68E* coding sequences not showing ambiguous positions. (.pdf, 94 KB)
- Figure S2 - Phylogenetic relationships of all gene sequences identified in a blastx search using the *D. melanogaster Muc68E* protein as the query and as database the predicted coding sequences for *Drosophila* species for which there is an annotation. (.pdf, 131 KB)
- File S1 - FASTA file with the alignment of the concatenated sequences of the 16 highly conserved genes used for the phylogenetic reconstruction of the 24 *Drosophila* species for which there is an available genome. (.txt, 299 KB)
- File S2 - FASTA file with the manual annotations of *Muc68E* based on the protein sequence of *D. melanogaster*. (.txt, 51 KB)
- File S3 - Alignment of the *Muc68E* coding sequences not showing ambiguous positions (FASTA format). (.txt, 50 KB)
- File S4 - Alignment of the mucin genes identified using blastx (FASTA format). (.txt, 130 KB)
