# Supplementary material for: The Drosophila melanogaster Muc68E Mucin Gene Influences Adult Size, Starvation Tolerance, and Cold Recovery
Source: G3 (Bethesda). 2016 Apr 25;6(7):1841–51. doi: 10.1534/g3.116.029934 (PMC4938639; doi:10.1534/g3.116.029934)
Supplement: Supplemental Material [file supp_g3.116.029934_FigureS1.pdf]

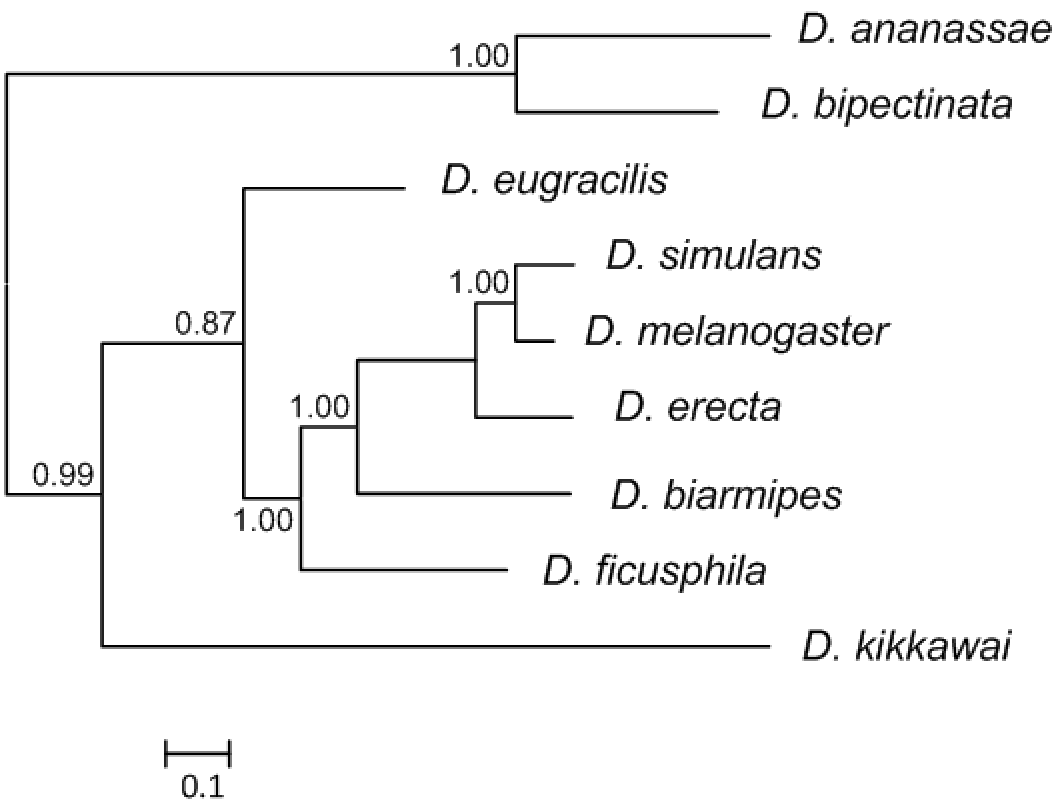


Fig. S1. Phylogenetic relationships of the annotated *Muc68E* coding sequences not showing ambiguous positions. Numbers near the nodes are posterior credibility values.
