# Supplementary material for: The Drosophila melanogaster Muc68E Mucin Gene Influences Adult Size, Starvation Tolerance, and Cold Recovery
Source: G3 (Bethesda). 2016 Apr 25;6(7):1841–51. doi: 10.1534/g3.116.029934 (PMC4938639; doi:10.1534/g3.116.029934)
Supplement: Supplemental Material [file supp_g3.116.029934_FigureS2.pdf]

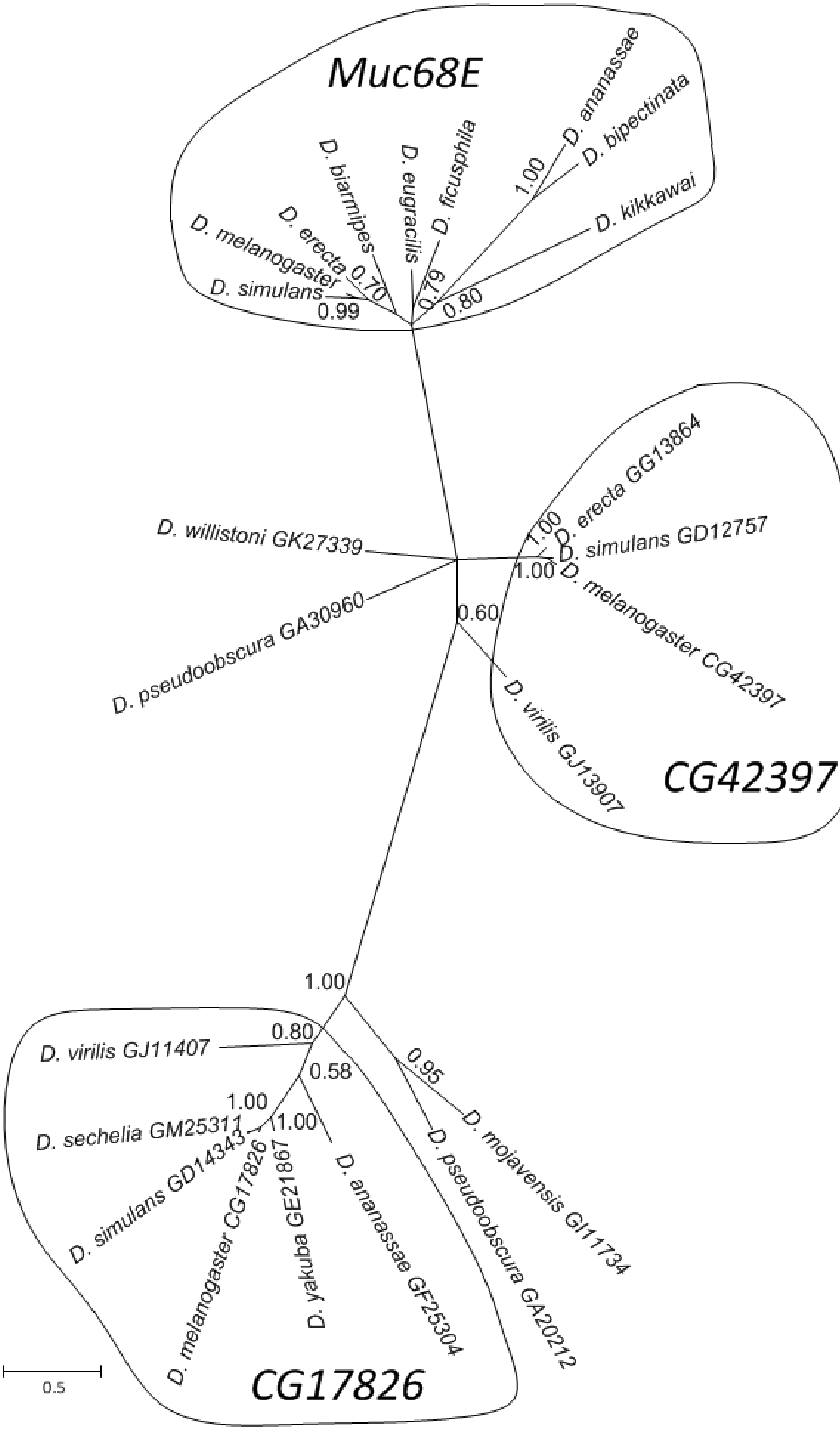
Muc68E
D. ananassae
D. bipectinata
D. kikkawai
D. ficusphila
D. eugracilis
D. biarmipes
D. erecta
D. melanogaster
D. simulans
1.00
0.70
0.79
0.80
0.99
D. willistoni GK27339
D. pseudoobscura GA30960
D. erecta GG13864
D. simulans GD12757
D. melanogaster CG42397
D. virilis GJ13907
1.00
1.00
0.60
CG42397
1.00
D. virilis GJ11407
0.80
0.58
0.95
1.00
1.00
D. sechelia GM25311
D. simulans GD14343
D. melanogaster CG17826
D. yakuba GE21867
D. ananassae GF25304
D. pseudoobscura GA20212
D. mojavensis GI11734
0.5
CG17826

Fig. S2 Phylogenetic relationships of all gene sequences identified in a blastx search using the *D. melanogaster Muc68E* protein as the query and as database the predicted coding sequences for *Drosophila* species for which there is an annotation. Numbers near the nodes are posterior credibility values. Circled genes are those recognized by Flybase (http://flybase.org) as being orthologous.
